# Cognitive Factors as Determinants of Typhoon Preparedness among Public High School Students in the Philippines

**DOI:** 10.4314/ejhs.v31i4.16

**Published:** 2021-07

**Authors:** Samson Mideksa

**Affiliations:** 1 Knowledge Translation Directorate, Ethiopian Public Health Institute, Addis Ababa, Ethiopia

**Keywords:** Cognitive (perceived severity, susceptibility, selfefficacy, and response efficacy), Typhoon preparedness, Public high schools' students, Philippines

## Abstract

**Background:**

Typhoon is the most common disaster in the Philippines and lead to injury, death, and damage to property. Although public hazard education is conducted with considerable effort, the disaster preparedness of society is low. Thus, this study aimed to assess the cognitive factors of typhoon preparedness among public high school students in the Philippines.

**Method:**

A descriptive correlation design was used to determine the relationship between cognitive with typhoon preparedness of grades nine and ten public high schools' students. Nine hundred thirty-three students were selected through purposive sampling from disaster prone areas of the Philippines.

**Result:**

The grand mean and standard deviation (M = 3.03, SD = .338) result revealed that the respondents' cognitive factors in terms of perceived severity, susceptibility, self-efficacy, and response efficacy toward typhoon preparedness (M = 3.11, SD = .421) was high. The result of Pearson's r, p-value and determination coefficient showed that perceived severity, selfefficacy, and response efficacy were positively linear in the relationship to planning, mitigation and response.

**Conclusion:**

The respondent's cognitive factors were high toward typhoon preparedness. Cognitive factors in terms of perceived severity, self-efficacy, and response efficacy determine typhoon preparedness.

## Introduction

A disaster is an event that occurs beyond the control or response mechanism of either the individual or the community. The most frequent disaster in the Philippines is the typhoon, most likely leading to injury, death and property damage (1,2). The people affected by these are not able to prepare or even survive (3). However, personal preparedness for food, water, first aid kits and medicine, sanitation and environmental needs and materials for entertainment for at least three days is basic (4). Typhoon Haiyan known as Typhoon Yolanda locally was one of the strongest typhoons that were ever recorded. It killed more than 7,300 people and 1.2 million people were evacuated in 2013 (5). Children and young people are seen as among the most vulnerable during disasters. They are disoriented, traumatized, and shocked. Similarly, they are forced to cope with separation from their families, the loss of their loved-ones, and the scarcity of food (6).

Cognitive factors refer to perceived severity, susceptibility, self-efficacy, and response efficacy. Perceived severity is an individual's perceptions to the extent of severity of a threat (7). Yang's study utilized tenth grade students and an open questionnaire to explore their ideas about flood. Most of the students were worried (8). In addition, perceived susceptibility is an individual's perception of how he or she is vulnerable to the threat. Weinstein as cited in Jorand informs that the greater the perceived susceptibility, the more preventive actions the individual will take to avoid the situation. In the same way, perceived self-efficacy is the individual's perception in evaluating protective measures with respect to threat and the ability to perform the action (9). The study finds that beliefs of self-efficacy have a direct influence on risk reduction behaviors (10). As well, perceived response efficacy is the feeling in which the level of confidence that the recommended preparedness measure helps the individual to be capable of protecting him/herself (7).

Disaster preparedness helps people to know what to expect and what to do in the scenario. When minutes count, it's important to know how to handle an emergency situation (4). Disasters come when least expected. Filipinos know that they are at risk but they are not making disaster preparedness a priority until they experience disaster (11). Based on this scenario, the researcher is interested to know cognitive factors including perceived severity, susceptibility, self-efficacy, and response efficacy towards typhoon preparedness among high school Filipino students.

## Methods

**Source**: The study was conducted in selected grade nine and 10 public high school students from Luzon, Visayas, and Mindanao in the Philippines.

**Study design**: This study used the descriptive correlational design to describe the cognitive (perceived severity, perceived susceptibility, perceived self-efficacy & perceived response efficacy) in correlation with disaster preparedness (planning, mitigation & response) among the students.

**Study population**: The study utilized purposive sampling from Luzon, Visayas, and Mindanao. International Institute of Rural Reconstruction (IIRR) Program Manager of Disaster Risk Reduction predicted typhoon prone areas of the Philippines. The researcher listed all public high schools in the specific areas. To choose the specific public schools, the random sample technique was utilized to select two schools from Cavite (Noveleta & Rosario), two schools from Tacloban (Tacloban city & Sagkahan) and one school from Davao (Catulanan Grande). A self-constructed questionnaire based on literature and related studies were used. Data were collected from 933 respondents during October 19, 2015 to November 27, 2015.

**Inclusion and exclusion criteria**: All students in the selected public high school who were enrolled for grade nine and 10 during data collection, and residing in the area for at least six months were recruited. Students who feel discomfort to respond, transferred to the area/school within six months, and absent during data collection were excluded from the study.

**Data processing and analysis**: The data gathered were encoded using Microsoft Excel and exported for analysis using the Statistical Package for Social Science (SPSS) version 22. Four-scale measurements (strongly disagree to strongly agree) were utilized to descriptive statistics such as frequency distribution, mean, and standard deviation to determine the level of cognitive factors on typhoon preparedness. To know the significant relationships between the cognitive factors on disaster preparedness, Pearson's product-moment correlation coefficient analysis of variance (r) was used.

**Ethical clearance**: Endorsement letters were secured from the Assistant Vice President for Academics-Graduate Studies to seek permission to gather data from the selected schools. The said letters were personally delivered to the respective division superintendents. The division superintendents then wrote a letter of endorsement to the principals of the selected schools. The researcher explained the purpose of the study, the nature of the questionnaire, the ethical issues involved and the inclusion criteria of the respondents to the respective principals, the coordinators of the schools and the classroom teachers. The purpose of the study was clearly explained to the participants. They were informed that participation was voluntary and they have the free will either to participate or not. The researcher also ensured the confidentiality of the respondents by letting them answer the questionnaires without writing their names and was assured that only the researcher has access to the questionnaires. The researcher emphasized to all respondents the importance of filing out all items of the questionnaire.

## Results

As shown in Table 1, the grand mean 3.15 and the standard deviation 0.55, reveals that the majority of the respondents highly perceived the severity of the impact of typhoon. Similarly, they agreed that they were susceptible to typhoon impact, as indicated by a grand mean of 2.94 and a standard deviation of 0.62. The perceived self-efficacy was also high since the grand mean was 2.84. As well, the respondents have high perceived response efficacy as shown in the grand mean of 3.18.

**Table 1:** Cognitive factors in terms of perceived severity, susceptibility, self-efficacy and Response efficacy of the Respondents in the Philippines, November 2015

No	Items	M	SD	SR	VI
1	Perceived Severity	3.1485	.54942	Agree	High
2	Perceived Susceptibility	2.9351	.6163	Agree	High
3	Perceived Self-efficacy	2.8416	.45732	Agree	High
4	Perceived Response Efficacy	3.1821	.50535	Agree	High

Scale: 1 – 1.49 (SD) = Very low, 1.50 – 2.49 (D) = Low, 2.50 – 3.49 (A) = High, 3.50 – 4 (SA) = Very high

The result reveals a grand mean of 3.16 which shows that the respondents were planning for the coming typhoon. As well, they had respondents have basic necessities at hand in preparation for the coming typhoon, as seen in the grand mean 2.89. In addition, the grand mean of 3.17, where the respondents agreed that they will respond to the coming typhoon (Table 2).

**Table 2:** Planning of the Respondents towards typhoon preparedness in the Philippines, November, 2015

No	Items	M	SD	SR	VI
1	Planning	3.1597	.49215	Agree	High
2	Mitigation	2.89	.793	Agree	High
3	Response	3.1710	.45346	Agree	High

Scale: 1 – 1.49 (SD) = Very low, 1.50 – 2.49 (D) = Low, 2.50 – 3.49 (A) = High, 3.50 – 4 (SA) = Very high

The result presented in Table 3 shows the relationship between the four dimensions of cognitive and planning of the respondents. Perceived severity has a positive significant linear relationship with the planning of the respondents, with r = .355 and *p*< 0.05. The coefficient of determination is .126025, which means that 12.6% of the variance in planning is accounted for by perceived severity. Perceived self-efficacy has a significant positive linear relationship of r = .402 and *p*< 0.05 with planning. The coefficient of determination is .161604, which means that 16.16% of the variance in planning is accounted for by perceived self-efficacy. Perceived response efficacy has a significant positive linearrelationshipr = .543 and a *p*< 0.05 with planning. Similarly, the coefficient of determination is .294849, which means that 29.48% of the variance in planning is accounted for by perceived response efficacy.

**Table 3:** Relationship between cognitive in terms of perceived severity, susceptibility, self-efficacy and response efficacy of the respondents, towards Planning in the Philippines, November 2015

	Pearson-r	r ^2^	*Significance*	Interpretation
Perceived Severity	.355**	.126025	.000	Significant
Perceived Susceptibility	.004	.000016	.896	Not significant
Perceived Self-Efficacy	.402**	.161604	.000	Significant
Perceived Response Efficacy	.543**	.294849	.000	Significant

** Correlation is s significant at the 0.05 level (2-tailed)

As indicated in the Table 4, the relationship between cognitive processes and the mitigation of the respondents. The perceived severity has a significant positive linear relationship of r = .211 and *p*< 0.05 with mitigation. The coefficient of determination is .044521, which means that 4.45% of the variance in mitigation is accounted for by perceived severity. There was a significant (r =.403 & *p*< 0.05) positive linear relationship between perceived self-efficacy and mitigation. The coefficient of determination is .162409, which means that 16.24% of the variance in mitigation is accounted for by perceived self-efficacy. In the same way, the perceived response efficacy has a significant positive linear relationship of r = .384 and *p*<0.05 with mitigation. The coefficient of determination is .147456, which means that 14.74% of the variance in mitigation is accounted for by perceived response efficacy.

**Table 4:** Relationship between cognitive in terms of perceived severity, susceptibility, self-efficacy and response efficacy of the respondents, towards Mitigation in the Philippines, November 2015

	Pearson-r	r^2^	*Significance*	Interpretation
Perceived Severity	.211**	.044521	.000	Significant
Perceived Susceptibility	-.028	.000784	.398	Not significant
Perceived Self-Efficacy	.403**	.162409	.000	Significant
Perceived Response Efficacy	.384**	.147456	.000	Significant

** Correlation is s significant at the 0.05 level (2-tailed)

The result presented in table 5 shows the relationship between the cognitive processes and the responses of the respondents. The perceived severity has a significant positive linearrelationship of r = .360 and *p*< 0.05. The coefficient of determination is .1296, which means that 12.96% of the variance in response is accounted for by its perceived severity. There was a significant (r =.362 & *p*< 0.05) positive linear relationship between perceived selfefficacy and response. The coefficient of determination is .131044, which means that 13.10% of the variance in response is accounted for by perceived self-efficacy. In the same way, perceived response efficacy has a significant positive linear relationship of r = .489 and *p*< 0.05 in response. The coefficient of determination is .239121, which means that 23.91% of the variance in response is accounted for by the perceived response efficacy.

## Discussion

The respondents had highly perceived the severity, perceived susceptibility, perceived self-efficacy, and perceived response efficacy. The respondents highly perceived the severity of the impact of the typhoon. This implies that a strong typhoon causes loss and damage to property and life. This result supports the findings of Blue Water Media; and Freitas; which states that typhoons kill many people, destroy houses, business sectors and schools (6,12). The study done by Lee, Ha, Kim, and Kwon, shows that there is high (67%) fear among elementary school students after typhoon Rusa in South Korean (13). As well, the respondents have a high perception of vulnerability to typhoon threat. This implies that the respondents perceived that the strong typhoon affects them and their properties. This is in line with the study of Peek and Fothergill, which states the poor have greater risk perceptions since they have little control over their lives. They worry more for their belongings than the high-income group (14). The respondent's perceived selfefficacy was high. This shows the respondents agreed to use the recommended preventive behavior in typhoon preparedness. This implies that the respondents influence their family in preparation for the coming typhoon, know what items to include in the emergency bag and where to go if they need evacuation. They develop strategies for the coming typhoon, are able to call their family whenever they feel so and are confident on what to do if the typhoon strikes. These results are supported by Marano, Geraci, and Legge study that stated people's beliefs about their capabilities to exercise control over events affect the way they live their lives (15). Perceived self-efficacy also sustains and regulates the cognitive, motivational and affective processes. In line with these, the respondents have high perceived response efficacy. This indicates that the respondents agree with the effectiveness of the recommended preventive behavior towards typhoon preparedness. This implies that the respondents were listening to the latest information about the typhoon from Philippine Atmospheric, Geophysical and Astronomical Services Administration (PAGASA). They also follow the instructions and recommendations of authorities like preparing emergency bags in case they need to evacuate. These results agree with that of Campasano's study which recommended preparedness measures to help the individual to be capable of protecting the self (7). On top of that, when the family teaches their children in advance about typhoons, the children become alert and more sensitive to the information. They tend to follow the information from television and hear the warning or instruction clearly (16).

The relationship between the perceived severity, perceived self-efficacy and perceived response efficacy has a positive significant linear relationship with disaster preparedness in terms of planning, mitigation, and response of the respondents. This result is in line with Rogers study as cited in Camposano which suggests that the exposure to threat communication will assess the individual's severity to the threat and the ability to perform the recommended protective actions and its effectiveness (7). The high perceived threat creates in the individual the desire to develop danger/fear circumstance, which leads to perceived efficacy. Danger control process which can be adjusted and intended for meeting the threat challenge can be the effect of a high level of perceived efficacy. According to Federal Emergency Management Agency (FEMA), response efficacy means danger control through preparedness with an effective response like the preparation of a disaster kit before the event in order to be a solution to the threat (17).

Based on the findings, it was concluded that the respondents were highly knowledgeable towards typhoon preparedness in terms of planning, mitigation and response. The higher the perception of the threat or severity, perceived self-efficacy, and response efficacy, the more they think and organize activity to achieve a desired goal in preparation for the coming typhoon. Although the study result shows a good result of the respondents in terms of typhoon preparedness, there is still room to improve their cognitive level. The respondents have to feel confident about what to do when there is a typhoon. Thus, health educators and teachers must continue on disseminating information regarding disaster preparedness to strengthen the skills thus enabling the parents, the community members enough time to plan, mitigate and respond to disasters.
